# Green synthesis of Fe and Zn-NPs, phytochemistry and pharmacological evaluation of *Phlomis cashmeriana* Royle ex Benth

**DOI:** 10.1016/j.heliyon.2024.e33327

**Published:** 2024-06-22

**Authors:** Amjad Hussain, Sajjad Azam, Kanwal Rehman, Meher Ali, Muhammad Sajid Hamid Akash, Xuefeng Zhou, Abdur Rauf, Abdulrahman Alshammari, Norah A. Albekairi, Abdullah Hamed AL-Ghamdi, Ahmad Kaleem Quresh, Shoaib Khan, Muhammad Usman Khan

**Affiliations:** a Institute of Chemistry, University of Okara, Okara, 56300, Punjab, Pakistan; b Department of Pharmacy, The Women University, Multan, Pakistan; c Department of Chemistry, Karakoram International University, Gilgit, 15100, Pakistan; d Department of Pharmaceutical Chemistry, Government College University, Faisalabad, Pakistan; e CAS Key Laboratory of Tropical Marine Bio-resources and Ecology, Guangdong Key Laboratory of Marine Materia Medica, South China Sea Institute of Oceanology, Chinese Academy of Sciences, Guangzhou, 510301, China; f Department of Chemistry, University of Swabi, Swabi, Pakistan; g Department of Pharmacology and Toxicology, College of Pharmacy, King Saud University, Post Box 2455, Riyadh, 11451, Saudi Arabia; h Pharmaceutical Care Department, Namerah General Hospital, Ministry of Health, Namerah, 65439, Saudi Arabia; i Department of Chemistry, University of Sahiwal, Sahiwal, 574000, Punjab, Pakistan; j Department of chemistry, Abbottabad University of Science and Technology AUST, Havelian, Abbottabad, Pakistan

**Keywords:** Green synthesis, Phytoconstituents, Cytotoxicity, Anti-inflammation, Anti-thrombolytic activity, Antibacterial potential

## Abstract

This investigation portrays the phytochemical screening, green synthesis, characterization of Fe and Zn nanoparticles, their antibacterial, anti-inflammation, cytotoxicity, and anti-thrombolytic activities. Four dissimilar solvents such as, *n*-hexane, chloroform, ethyl acetate and *n*-butanol were used to prepare the extracts of *Phlomis cashmeriana* Royle ex Benth. This is valued medicinal plant (Family Lamiaceae), native to mountains of Afghanistan and Kashmir. In the GC-MS study of its extract, the identified phytoconstituents have different nature such as terpenoids, alcohol and esters. The synthesized nanoparticles were characterized by SEM, UV, XRD, and FT-IR. The phytochemical analysis showed that the plant contains TPC (total phenolic content) 297.51 mg GAE/g and TFC (total flavonoid content) 467.24 mg CE/g. The cytotoxicity values have shown that the chloroform, *n*-butanol and aqueous extracts were more toxic than other extracts. The anti-inflammatory potential of *n*-butanol and aqueous extracts was found higher than all other extracts. Chloroform and *n*-hexane extracts have low MIC values against both *E. coli* and *S. aureus* bacterial strains. Chloroform and aqueous extracts have great anti-thrombolytic potential than all other extracts. Overall, this study successfully synthesized the nanoparticles and provides evidence that *P. cashmeriana* have promising bioactive compounds that could serve as potential source in the drug formulation.

## 1 Introduction

Nanotechnology has lately emerged as one of the most important science due to technical improvement in a wide range of study fields, for example chemistry, biology, physics, material science, pharmacy, environment, and medicines [1]. The utilization and control of matter in such a way that one of its dimensions founds within 1–100 nm range is known as nanotechnology [2]. Richard Feynman initially introduced the concept of nanotechnology at the American Physical Society's (APS) annual meeting at the time of 1959. Professor Norio Taniguchi (Tokyo Science University) in 1974, elaborate the word “nanotechnology” to describe the particular formation of materials [3]. Because of their shape, composition, specific size, higher surface area, and purity of individual ingredients, nanoparticles have various unique features. They can be employed as nanomagnets, water disinfectants, drug/gene delivery systems, and in electrical devices as a quantum dots, catalysts, and contamination remediation agents due to their characteristics [4,5]. This effectiveness of nanoparticles is because of their unique synthesis method. Even little changes in the synthesis method can result in significant changes in their fundamental characteristics.

Nanoparticles could be produced through number of techniques. In the formation of nanoparticles, a wide range of physico–chemical techniques are now being used (NPs). The formation of metal nanoparticles by using reducing agent from the plant source is a cheaper, chemically pure, and environment friendly method in the field of biology and medicines. Biological reduction is a valuable strategy comparable to chemically reduction in which a chemical is replaced with a natural product-extract through growth terminating, capping and stabilizing capabilities [6]. Both chemical and physical ways of synthesis are costly and produce harmful side products.

The biologic technique, on the other side, is inexpensive, simple in formation, lowers chemically pollution, and removes needless processing throughout the synthesis process [7]. Furthermore, the fact has been established that physical and chemical procedures have uncertainties in their size, dispersion of nanomaterials, shape and most importantly, the utilization of expensive and harmful reducing agents, all of which end up in the surrounding, increasing pollution. So, the formation of metal nanoparticles through the biosynthesized method is developing day after day.

The simplest, most economical and reproducible method is the green synthesis of metallic nanoparticles using various plant parts, such as the leaf, stems, seed, and root. The natural composition of many organic reducing compounds found in plants makes them a preferred option for producing nanomaterial, since they can readily adapt to the synthesis of nanoparticles. Higher antioxidants found in seeds, fruits, leaves, and stems as phytochemical constituents are obtained by various herbs and plant sources. Therefore, there is a significant synergy between natural/plant sciences and nanotechnology due to the use of phytochemicals derived from plants in the entire synthesis and construction of nanoparticles. The term “green nanotechnology” refers to this association's distinctively environmentally friendly approach to nanotechnology. Developing these production methods without contaminating the environment would establish new standards for clean and green technologies that are both extremely sustainable and economically feasible [[8], [9], [10]].

The morphology, size, and economical applications of the green synthesized Fe and Zn nanoparticles from the different plants sources are listed in Table 1 and Table 2 respectively.

**Table 1:** The characteristics of FeNPs synthesized from the different parts of Plants.

Plant source	Plant parts	Morphology	Size (nm)	Application	Ref.
*Dodonaea viscose*	Leaf	Spherical	50–60	Antibacterial	[11]
Green tea and *Eucalyptus*	Leaf	Quasi-spherical	20–80	Nitrates removal	[12]
*Eucalyptus*	Leaf	Amorphous	20–80	Treatment of eutrophic wastewater	[13]
Green-Tea	Leaf	–	–	Soil mineralogy	[14]
*S. jambos* (L.) Oolong tea, *A. moluccana* (L.)	Leaf	–	–	Removal of chromium	[15]
Green tea	Leaf	–	–	Transport properties of nano zero-valent iron (nZVI) through soil	[16]
Green tea	Leaf	Spherical	5–10	Removal of hexavalent chromium	[17]
*Eucalyptus globules*	Leaf	Spherical	50–80	Adsorption of hexavalent chromium	[18]
Green tea	Leaf	Spherical	70–80	Degradation of dye (malachite green)	[19]
Green tea	Leaf	–	20–120	Degradation of monochlorobenzene	[20]
*Salvia officinalis*	Leaf	Spherical	5–25	–	[21]
*Oolong tea*	Leaf	Spherical	40–50	Degradation of malachite green	[22]
*Aloe vera*	Leaf	Cubic crystalline	6–30	–	[23]
Green tea	Leaf	Crystalline	40–80	Photo catalytic activity	[24]
Orange extract	Peel	Cubic cystalline	30–50	–	[25]
Sorghum	Bran	Spherical	40–50	Degradation of bromothymol blue	[26]
Alfalfa	–	–	1–10	–	[27]
Alfalfa	–	–	<5	–	[28]
*Syzygium cumini*	Seed	Crystalline spherical	9–20	–	[29]
*Passiflora tripartitavar*.	Fruit	Spherical	18–24	–	[30]
*Terminalia chebula*	Fruit	Amorphous chain-like	<80	–	[31]
GarlicVine (*Mansoa alliacea*)	Leaf	Crystalline	13–15	–	[32]
*Hordeum vulgare* and *Rumex acetosa*	Leaf	Amorphous	10–40	–	[33]
*Punica granatum*	Leaf	–	100–200	Hexavalent chromium removal	[34]
*Tridax procumbens*	Leaf	Irregular sphere shape	80–100	Antibacterial	[35]
*Azadirachta Indica*	Leaf	Spherical	50–100	–	[36]
Carob	Leaf	Mono-dispersed crystalline	5–8	–	[37]
Grape	Leaf	Amorphous quasi-Spherical	15–100	Azo dyes such as acid Orange	[38]
*Eucalyptus tereticornis*, *Melaleuca nesophila*, and *Rosemarinus officinalis*	Leaf	Spherical	50–80	Catalyst for decolourisation of azo dyes	[39,40]
*Eucalyptus tereticornis*	Leaf	Cubic	40–60	Adsorption of azo dyes	[41]
*Azadirachta indica*	Leaf	–	100	–	[42]
Tea	Powder of tea	Spherical	40–50	–	[43]
Green tea	Leaf	Crytalliine spherical	70	–	[44]
Green tea	Leaf	Amorphous	40–60	Degradation of aqueous cationic and anionic dyes	[45]
*Camellia sinensis*	Leaf	Spherical	5–15	Bromothymol blue degradation (organic contamination)	[46]

**Table 2:** The characteristics of ZnNPs synthesized from the different parts of Plants.

Plant source	Plant parts	Morphology	Size (nm)	Ref.
*Laurus nobilis* L	Leaf	Hexagonal Wurtzite	25–26	[47]
*Catharanthus roseus*	Leaf	Hexagonal Wurtzite	50–90	[48]
*Cassia alata*	Leaf	Spherical	60–80	[49]
*Psidium guajava*	Leaf	Spherical	13–28	[50]
*Olea europaea*	Leaf	Spherical	11–28	[50]
*Ficus carica*	Leaf	Spherical	11–24	[50]
*Citrus limon* Osbec	Leaf	Spherical	11–24	[50]
*Pandanus odorifer*	Leaf	Spherical	90	[51]
*Matricaria chamomilla* L	Flowers	Crystalline	49–191	[52]
*Olea europaea*	Leaf	Crystalline	40–124	[52]
*Lycopersicon esculentum* M.	Fruits	Crystalline	65–133	[52]
*Coccinia abyssinica*	Tuber	Hexagonal	10.4	[53]
*Couroupita guianensis*	Leaf	Nanoflakes	–	[54]
*Euphorbia jatropa*	Latex	Hexagonal	6–21	[55]
*Nyctanthes arbor-tristis*	flowers	Spherical	12–32	[56]

Medicinal plants have received a lot of focus in recent decades because of their therapeutic effects on a variety of diseases [57,58]. These are the primary sources of important phytochemicals that are used in the development of novel medicines [57]. Alkaloids, flavonoids, phenolics, and terpenes, among other phytochemicals, have been evaluated to offer potential in the treatment of a variety of health problems, especially cancer prevention [59]. These chemicals also have a substantial anti-oxidant effect, since they help to reduce oxidative stress. Approximately 20,000 plants have been utilized to treat various ailments [60]. In Africa, for example, more than ¾ of the population relies on medicinal plants for traditional cures [61]. Medicinal plants have a long history of being used to treat a variety of ailments. These plants have provided a vital insight into traditional medicinal systems like Ayurvedic, Chinese, and Unani. Many medications have been developed as a result of these systems, and they are continuously being tested in various situations. Synthetically prepared medications have become the predominant source of medication in the developed countries [60,62]. Because of its side effects and microbial resistance to these medications, the research paradigm has shifted to ethnopharmacognosy [63]. The bioactive chemicals found in the medicinal plants are being used for therapeutic purposes or as a precursor in the production of pharmaceuticals [[64], [65], [66], [67]].

*Phlomis* is a medicinally significant genus that contains a wide range of bioactive chemicals [68]. It is a member of the Lamiaceae family, which has around 100 species. The majority of these species are found in Asia, Turkey, Europe, and North Africa [69,70]. Traditionally, aerial parts of *Phlomis* species are used to make herbal tea, which have been proved to be effective against stomach ulcers and hemorrhoids [71,72]. Many beneficial chemicals have been isolated from the genus *Phlomis*, including monoterpenes, sesquiterpenes, certain aliphatic compounds, phenyl thyl alcohol, iridoids and flavonoids [73] as per various phytochemical investigations [74]. These isolated chemicals have been discovered to be extremely active in a variety of pharmacological activities, including antibacterial activity [71], antioxidant [69], anticancerous [75], immuno-suppressive [76], anti-inflammatory [77] and free radical scavenging [78]. About 70 species of the genus *Phlomis* are still being studied phytochemically in the hopes of discovering novel plant-based lead sources [79].

According to our best knowledge, it is represented that there is no any evaluation of *Phlomis cashmeriana* in these biological activities before inspite of having valuable phytochemical compounds such as flavonoids and terpenoids from this plant species [80]. Hussain et al. (2010) found two different compounds which were isolated from the *P. cashmeriana*, namely, phlomisamide and stigmasterol [80]. In another study, they conducted survey on the same plant species and 12 known compounds were isolated for the first time, in which four compounds were flavonoids, five compounds were triterpenes, one shikimic acid deriative, and two compounds were steroids. Names of these 12 compounds were apigenin 7,4-dimethyl ether, luteoline-7-methyl ether, bitalgenin, kaempferol 3-*O*-3‴-acetyl-*α*-Larabinopyranosyl-(1‴-6‴)-*β*-d-glucopyranoside, glutinol, oleanolic acid, *β*-amyrin, ursolic acid, 3-*O*-*p*-coumaroylshikimic acid, 3*β*-hydroxycycloart-24-one, and a mixture of *β*-sitosterol, and stigmasterol [81]. Kashmir sage is the common name of *P. cashmeriana*, which is locally known as Darshol. Locally, the entire plant body is used to cure bone fractures [82]. Dirty lands and sloppy regions are the habitats of this species. *P. cashmeriana* is a plant native to the Himalayan region [81], extensively growing in the areas between Afghanistan and Kashmir.

In the current study, an effort has been made to explore (i) the phytochemical information of *P. cashmeriana* for further designing a new drug from the identified phytochemicals, and (ii) In vitro examination for the antibacterial, anti-inflammation, anti-thrombolytic and cytotoxicity properties. This study provides combinations of new anti-thrombolytic and antibacterial, cytotoxic and anti-inflammatory medications in comparison with many medicinal plants and further in vivo study may reveal it as a good candidate for treating various ailments.

## 2 Experimental

### 2.1 Collection and preparation of plant material

Fresh plant species was collected from the mountains of Malikhel village; District Kurram, N.W.F.P Pakistan, in December 2020. This plant was dried under shaded area, and then grinded into powder form weighting 7.5 kg through using blander and placed in the plastic bag at the 25 °C temperature.

### 2.2 Formation of plant extraction

The plant powder was soaked in 95 % CH_3_OH for 12 d (3 × ) at normal temperature then filtered. The methanol was evaporated in a Scilogex Re-100 pro rotary evaporator (Starlitech) and crude extract was placed in the fuming hood for further concentration. The obtained crude extract weighting 1.25 kg was dissolved in distilled water. By following sequential extraction method, five sub fractions were obtained such as *n*-hexane (PCH), chloroform (PCD), ethyl acetate (PCE), *n*-butanol (PCB) and aqueous fractions (PCA) in 10.81 % (135.15 g), 1.09 % (13.62 g), 1.03 % (12.83 g), 7.85 % (98.12 g), and 79.2 % (990 g) yields respectively.

### 2.3 GC-MS analysis of crude extract of Phlomis cashmeriana

GC–MS study of the crude extract (PC) of *Phlomis cashmeriana* was performed by means of Agilent-Technologies 8860 gas chromatographic (GC) equipment, coupled with the Agilent 5977B MSD from the CLC Food Chemistry Lab, UVAS, Pakistan. Capillary column HP-5ms (30 m × 0.25 mm with thickness film 0.25 μm) were used for isolation of compounds. The temperature of the detector and injector were set at 280 °C. The oven temperature was held at 80 °C for 1 min, then increasingly it ramped at 40 °C/min to 120 °C where it keep constant for 2 min then finally kept it 310 °C for 10 min. Helium gas with 50 mL/min at 0.7 mints flow rate was used as carrier gas. PC was diluted with the methanol solvent. Pulse splitless mode is used for sample injection. Ionization energy of 70 eV has used in mass spectrometer for the detection. Comparing the spectrum with (NIST 05 library) and contrasting their relative retention times with previous literature data of compounds have evaluated.

### 2.4 Green synthesis

#### 2.4.1 Stock solution

0.01 M of FeCl_3_.6H_2_0 and ZnCl_2_ stock solutions were prepared in distilled water for the formation of nanoparticles. All these chemicals were obtained from Sigma-Aldrich.

#### 2.4.2 Preparation of nanoparticles

Iron and Zinc nanoparticles were produced by using crude extract of *P. cashmeriana* through an easy and conventional heating method. For nanoparticles preparation, 0.5 mL of crude extract was taken in 50 mL of distilled water in separate two flasks and then resulting solutions were stirred with magnetic stirrer for 1 h. One flask mixed with 5 mL of stock solution of FeCl_3_.6H_2_O, and other with 5 mL of stock solution of ZnCl_3_. The resulting mixtures were stirred at 70 ^ᵒ^C for 1 h. After each 5-min, the temperature difference was observed. After the solution had cooled, the products were separated by 80-1 centrifuge machine (China) for 2–3 min at 10,000 rpm. The resulting products were dried for 3 h at room temperature. In the formation of iron and zinc nanoparticles, the crude extracts (filtrate) functions as a reducing, capping, and stabilizing agent [83].

### 2.5 SEM

When evaluating nanoparticles physically, the most stable nanoparticles are considered to be the most suitable ones. The digital images of the surface morphology were obtained using scanning electron microscopy (SEM) (JEOL, JSM-6400, Japan) with a secondary electron detector at an accelerating voltage of 15 kV. For this, solid nanoparticles of iron and zinc salts that were produced through the green synthesis from a crude extract of *P. cashmeriana* were employed on SEM grid. The sizes of nanoparticles in obtained SEM images were calculated by using ImageJ software.

### 2.6 UV analysis

At room temperature, UV visible spectra were recorded by BK-D560 spectrophotometer (China) to monitor the synthesis and stability of Fe-NPs and Zn-NPs. The base line was drawn by the methanol solution, and the wave length (λ) was set between 250 nm and 800 nm. The synthesis of Fe and Zn-NPs are indicated by the peaks between the 250–350 nm and 300–400 nm ranges respectively.

### 2.7 XRD analysis

Using a high-power Cu-Kα radioactive source (λ = 0.154 nm) at 40 kV/40 mA, X-ray diffraction (XRD) patterns of Fe and Zn-NPs with MG were produced using a Philips-X'Pert Pro MPD (Netherlands). From 10° to 80° 2θ, these green synthesized nanoparticles were scanned at a rate of 3° 2θ per minute. This scanning range covered all the required species of Fe, Zn and their oxides.

### 2.8 FT-IR analysis

The FT-IR study of the crude extract of *P. cashmeriana*, Fe and Zn-nanoparticles were studied by IRSpirit with QATR-S Mounted (single-reflection ATR accessory with a diamond crystal).

### 2.9 Total phenolic components

The total phenolic components were estimated by following Julkenen-Titto method [84]. In this assay, precisely 100 μL of methanol-dissolved crude extract (1 mg/mL) were subjected into test tube with 1 mL of Folin-Ciocalteu reagent and then added distilled water for final volume completion up to 10 mL. The mixture was allowed to incubate for 5 min then vortex mixed at two times at room temperature. Then, 2 mL of sodium carbonate Na_2_CO_3_ (7.5 %) has added to the mixture and standing in the shade for 2 h. The sample absorbance was read at 765 nm by using an ultra-violet spectrophotometer (Lambda 25, PerkinElmer, USA). The Gallic acid was taken as standard with R^2^ value 0.99.

### 2.10 Total flavonoids components

Aluminium chloride colorimeter assay was used for the determination of the flavonoids compound from the sample (PC). An aliquot almost 1 mL of the sample i.e. crude extract or the standard solution of Catechin in 100, 80, 60, 40, 20 mg/L was taken into volumetric flask of 10 mL which already consisting distilled H_2_O (4 mL). After this, added 0.3 mL of 5 % of sodium nitrite. After the time of 5 min, the 0.3 mL of 10 % aluminium chloride (AlCl_3_) was included. Then after the 1 mint, 2 mL of the 1 M sodium hydroxide (NaOH) was taken in the flask and raise the volume up to 10 mL with distilled H_2_O. Then whole solution was shaken and subjected into UV for the determination of absorbance at 510 nm. The flavonoids component of crude extract was measured in mg CE (catechin equivalents) per gram of crude extract with R^2^ value 0.9835 [85].

### 2.11 In vitro biological assays

#### 2.11.1 Hemolytic assay

The hemolytic assay was used to evaluate the cytotoxic potential of PCH, PCD, PCE, PCB, PCA, Fe and Zn NPs on the viscous pellets which obtained after the centrifugation of 3 mL of human blood cells. In this process, 3 mL blood of healthy human was taken in sterile polystyrene screw-cap tube of 15 mL size and centrifuges for the time of 15 min. The resulted pellet cells in microfuge vessels were washed with chilled (3–4 ^ᵒ^C) sterile isotonic PBS for standardization and incubated for 24h under control standard conditions. The resulted suspension was diluted with sterile PBS up to 7.1 × 10^8^ cells/mL for the each tested assay. 0.1 % Triton X-100 and PBS were used as standards in each assay as a positive and negative control respectively. 20 μL aliquots of 250 μmol/L concentrated samples were placed aseptically in 2 mL microfuge vessels. Poured 180 μL blood viscous pallets in each 2 mL centrifuge tubes and placed each vessel in the incubation at 37 °C for 35 min, followed by centrifugation after cooling at 0 °C for 5 mints to get its supernatant. The tubes containing 100 μL of all the supernatant were placed on wet ice, after diluted with 900 μL cooled sterile PBS. After this, their UV absorbance was determined at 576 nm using Bausch and Lomb Spectronic 1001 spectrophotometer which resulted into percentage of toxicity values of all the samples [86].

#### 2.11.2 Anti-inflammatory activity

Williams et al. assay was used for the estimation of anti-inflammatory activities of the required samples. In this method, 500 μL BSA (bovine serum albumin) with 0.2 % (w/v) solution was prepared with tris-buffer saline and with a control pH of 6.74 by utilizing CH_3_COOH for all the tested samples. After formation of BSA, 5 μL of test compound/standard was added in it. The resulting mixture was heated for 5 mints at 72 °C. After that, the mixture was cool down at room temperature and subjected into UV spectrophotometer for measurement of absorbance at 660 nm. The control mixture was containing 500 μL of BSA and 5 μL of CH_3_OH. The percentage of anti-inflammatory potential was calculated by comparing of stabilization capacity of BSA with positive control Diclofenac and negative control DMSO with the tested samples [87].

#### 2.11.3 Minimum inhibitory concentration (MIC)

The MIC value was examined against the bacterial strain “*Escherichia coli* and *Staphylococcus aureus*”. Under aseptic circumstances, ELISA plates were prepared. The entire samples in different concentration were pipetted into the ELISA plate (disposable sterile polystyrene plates) with the quantity of 20 μL. Adding 50 μL of broth medium in it. Two-fold serial dilutions have done by using a pipette so which in serially decreasing concentration each well had 10 μL of the sample materials. At the end, 10 μL of microbial mixture (5 × 106 cfu/mL) added. ELISA plates were covered with para-film followed by incubation for 24 h at 37 °C for the growth of colonies of bacteria. The extent of growth of bacterial colonies told about the antibacterial potential of subjected samples [86].

#### 2.11.4 Thrombolytic activity

For the in-vitro study of clot lysis potential of subjected samples, blood of human healthy volunteers was taken in eppendorf tubes and allowed to clotting. The eppendorf tubes were placed in incubation on 37 °C for ¾ hour. The tubes were weighted after blood clotting. Each samples and standard compounds in 100 μL was taken in separate eppendorf tube consisting already weighted clotted blood and placed again in incubation at 37 °C for 90 mints. After this, clot lysis process was started. The serum was separated from the left clots in tubes. Weighted the left clots and compared this weight with previous weight of clots before reaction. This difference was determined the anti-thrombolytic potential of all samples. Streptokinase and water were taken as standards [88]. Following equation (1) was used for the calculation of the % of clot lysis.

(1) $$\%\;\text{of}\;\text{clot}\;\text{lysis}=\frac{\text{wt}.\;\text{of}\;\text{the}\;\text{lysis}\;\text{clot}}{\text{wt}.\;\text{of}\;\text{clot}\;\text{before}\;\text{lysis}}\times 10$$

## 3 Results and discussion

### 3.1 GC-MS analysis of methanolic crude extract

**Fig. 1:**
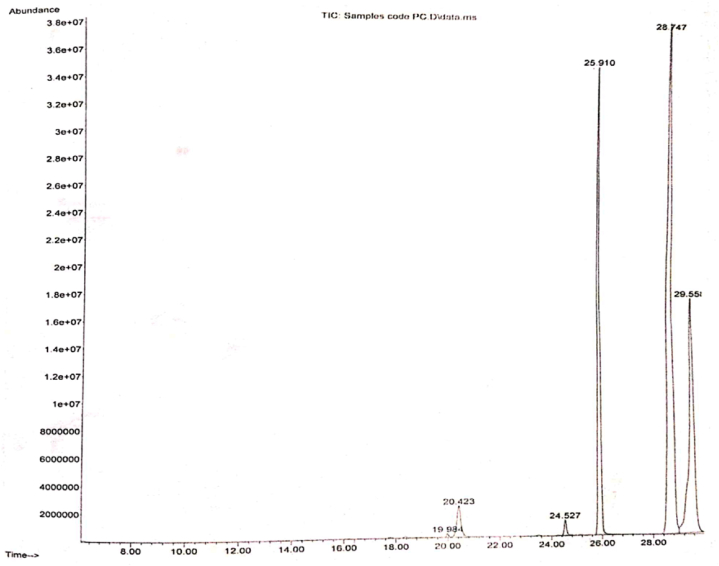
GC-MS analysis report for the extract of *P. cashmeriana*.

The findings of GC-MS analysis of crude extract (PC) are enlisted in Table 3. Six different compounds were identified from the GC-MS analysis of the crude extract of the *P. cashmeriana* that could be the responsible phytochemicals for the medicinal properties of this plant. The confirmations of these identified compounds were based on their particular retention times, molecular weight and their molecular formulas. The GC-MS chromatogram of *P. cashmeriana* is given in Fig. 1 and identified compounds are enlisted in Table 3.

**Table 3:** Chemical composition of *P. cashmeriana* methanolic extract.

Sr. no.	R/T	Compound name	‵Molecular formula	Mol. wt.	Peak area %
1	19.984	Silane, trimethyl [[5-methyl-2-(1-methylethyl) cyclohexyl]oxy]-	C_13_H_28_OSi	228.45	0.145
2	20.423	3-Methoxy-D-homoestra-1, 3, 5(10-trien-17a-one (8–9 & 14 = α)	C_20_H_25_O_2_	297.43	2.675
3	24.527	2-Cyclopenten-1-one, 2-pentyl-	C_10_H_16_O	152.23	0.754
4	25.910	1,4-Anthracenedione, 5,6,7,8-tetra hydro-2-methoxy-5,5-dimethyl-	C_17_H_18_O_3_	270.32	23.143
5	28.747	1,5,9-Cyclododecatriene, (*Z,Z,Z*)-	C_12_H_18_	162.27	48.511
6	29.558	6-Octadecenoic acid, methyl ester, (*Z*)-	C_19_H_36_O_2_	296.49	24.772

### 3.2 SEM analysis of green synthesis of Fe-NPs and Zn-NPs

**Fig. 2:**
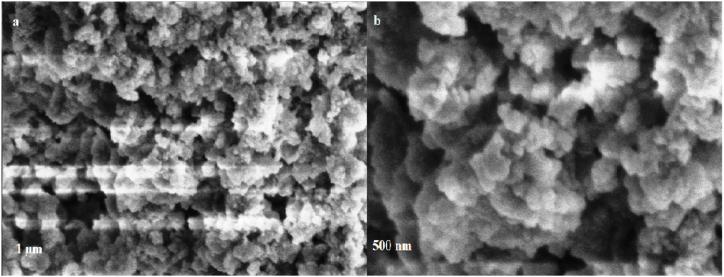
SEM images of Fe-NPs; a) low magnification, b) high magnification.

**Fig. 3:**
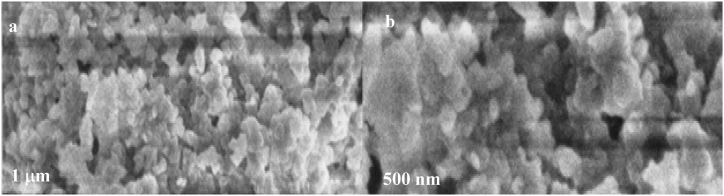
SEM images of Zn-NPs; a) low magnification, b) high magnification.

The effective synthesis of nanoparticles was revealed by the SEM images of Fe-NPs, and Zn-NPs which are displayed in Fig. 2 (a, b) and Fig. 3 (a, b) respectively. It is obvious that both of the particles are in nano-sized ranges and are appears in spheroidal with the average diameter of approximately 12 nm and spherical shapes with the average diameter of approximately 11 nm in case of Fe-NPs and Zn-NPs respectively. The nonuniform and bigger appearance of particles at certain positions may be attributed to adhesion and aggregation of individual particle during drying. However, the irregular cluster of these nanoparticles indicating that the polyphenols and flavonoids were responsible for their reducing and capping agent [19,89]. The extract of *P. cashmeriana* is consisting of different naturally occurring substances with various reducing properties which are responsible for the metal reductions. There are less aggregates in the green synthesized Fe-NPs, and Zn-NPs, despite the fact that the pure compounds are more effective at developing thin dispersed particles [90].

*P. cashmeriana* extract contains various phytoconstituent including flavonoids, terpenoids, and polyphenols or antioxidants which likely have a significant role in regulating the aggregation of the nanoparticles and enhance their dispersion by serving as a capping agent. With its high phenolic content and antioxidant capacity, *P. cashmeriana* extract might make a good option for synthesizing different metal nanoparticles.

### 3.3 UV–Vis spectroscopy

**Fig. 4:**
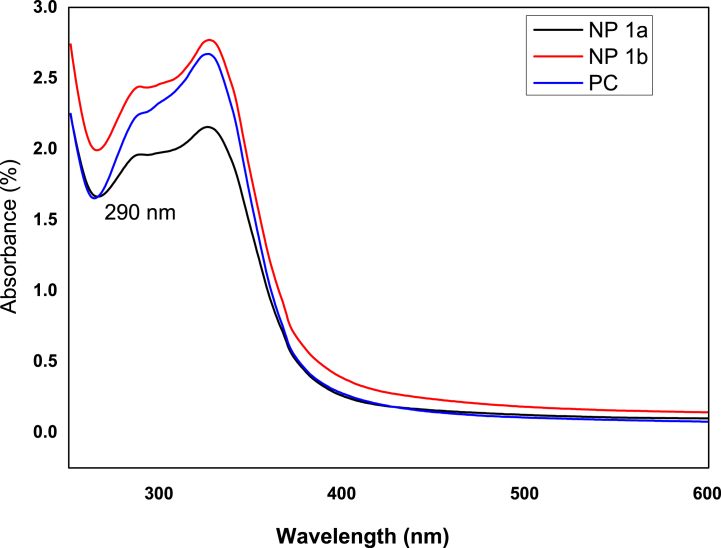
UV data for the crude extract (PC) and synthesized Fe-NPs. NP 1a: PC solution and salt solution in 1:1 v/v, NP 1b: PC solution and salt solution in 1:2 v/v.

**Fig. 5:**
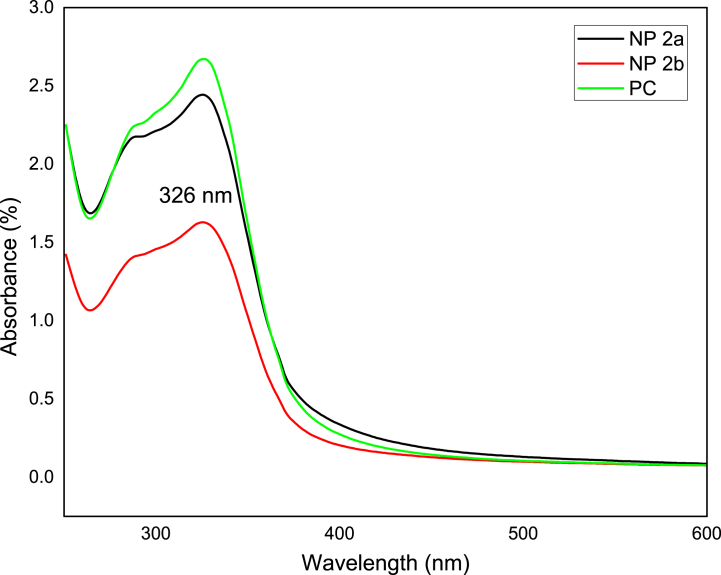
UV data for the crude extract (PC) and synthesized Zn-NPs, NP 2a: PC solution and salt solution in 1:1 v/v, NP 2b: PC solution and salt solution in 1:2 v/v.

UV–Vis spectroscopy, which particularly analyses the surface plasmon resonance peaks of nanoparticles, is typically used to detect the formation of Fe-NPs as well as Zn-NPs. Both the metals showed distinctive optical characteristics as a result of the surface plasmon resonance property. The development of Fe-NPs, and Zn-NPs were shown by the change in color of the salt solutions from green to dark brown and green to light orange respectively when extract was added at a temperature of 30–40 °C. The active phytochemicals in *P. cashmeriana* extract reduced iron (Fe^3+^ to Fe^0^) and Zn (Zn^2+^ to Zn^0^), which resulted in a change in the solution's colors. Earlier investigations indicate that phytochemicals function as capping and lowering agents. Natural extracts from a range of sources had a variety of components that were used to produce symmetrical nanoparticles. Stock solutions of *P. cashmeriana* extract and various salt concentrations were used in this experiment. The spectra of the synthesized Fe-NPs and Zn-NPs were measured against methanol to monitor the synthesis and stability of NPs. The spectra’ showed the peaks at 290 nm and 326 nm, which are specific for the Fe and Zn nanoparticles respectively. In methanol solutions, the highest absorbance of Fe-NPs and Zn-NPs were seen in the wavelength (λ) ranges of 250–350 nm, and 300–400 nm respectively indicating the successful production of Fe-NPs [91] (Figure-4) and Zn-NPs [92] (Figure-5).

### 3.4 XRD of Fe-NPs and Zn-NPs

**Fig. 6:**
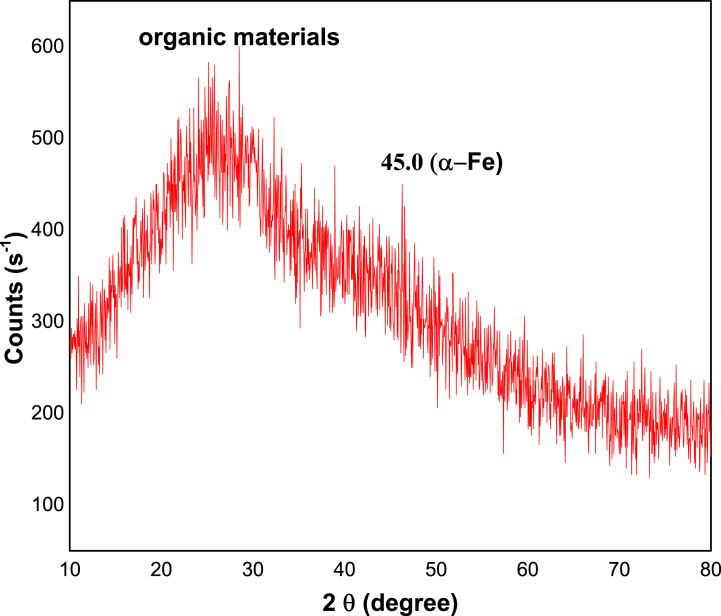
XRD pattern for the green synthesize Fe-NPs. (For interpretation of the references to color in this figure legend, the reader is referred to the Web version of this article.)

**Fig. 7:**
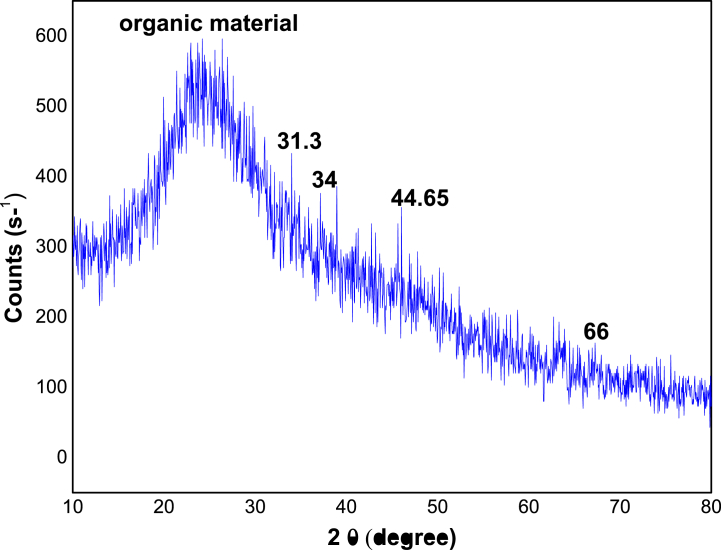
XRD pattern for the green synthesize Zn-NPs. (For interpretation of the references to color in this figure legend, the reader is referred to the Web version of this article.)

**Fig. 8:**
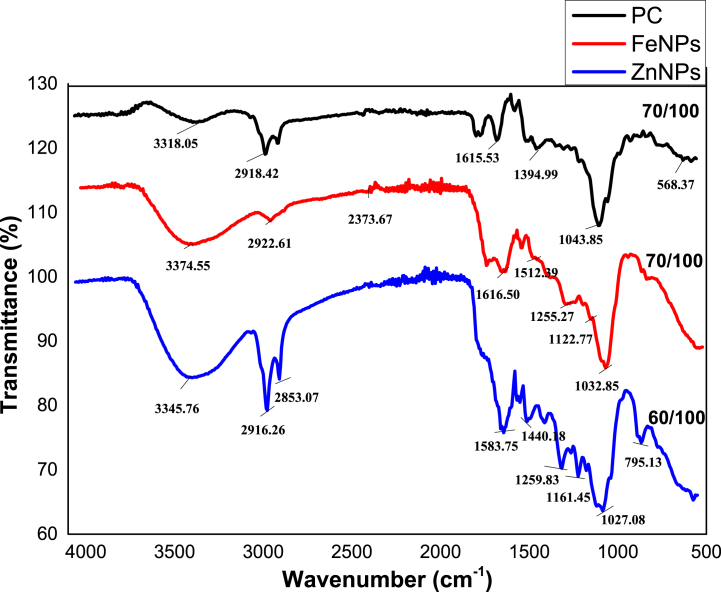
FT-IR spectrum of crude extract of *P. cashmeriana* (PC), FeNPs, and ZnNPs.

The XRD pattern of Fe-NPs and Zn-NPs are shown in Fig. 6, Fig. 7 respectively. It reveals that there are few distinguishable diffraction peaks throughout the entire pattern of both nanoparticles, indicating that the synthesized Fe-NPs and Zn-NPs are mostly amorphous in nature. In the case of Fe-NPs, roughly at 2θ of 45^ᵒ^, zero-valent iron (α-Fe) showed a less significant distinctive peak [13,93] Although in the case of Zn-NPs, some less significant indications are observed at 2θ of 31.3^ᵒ^, 31.7^ᵒ^, 34^ᵒ^, 44.65^ᵒ^, 66^ᵒ^, and 67.1^ᵒ^. The subsequent FTIR data in Fig. 8 was consistent with the broad shoulder peak in both XRD pattern at 2θ = 24° being organic compounds adsorbed from *P. cashmeriana* extract as a capping and stabilizing agent. When Fe-NPs were produced using *Terminalia chebula* aqueous extract, the pattern was identical [94]. This Zn-NPs pattern was related with the earlier study on nanoparticles formation by using *R. sativus* var. Longipinnatus leaf's extracts [95].

### 3.5 FT-IR analysis

The IR study is employed to find out the nature of pure extract, and green synthesized nanoparticles and also the presence of phytoconstituent in this plant extract. The presence of phytochemicals is responsible for the shape modification and stabilization of synthesized particles. The IR spectrum of crude extract (PC) showed a broad peak at frequency 3318.05 cm^−1^ indicated the presence of alcoholic group present (could be Phenolic OH). Other peak at frequency 2918.42 cm^−1^ showed the presence of carboxylic acid OH stretching and -C-H stretching also. Another peak at frequency 1615.53 cm^−1^ shown the presence of C=C of the aliphatic and C=C aromatic regions respectively. Peak at 1394.99 cm^−1^ indicated that there may be NO_2_ group presents. The % transmittance peak at frequency of 1043.85 and 568.37 cm^−1^ indicated the presence of alkyl halides (Figure-8).

In case of FT-IR analysis of Fe-nanoparticles, the frequency of 3374.55 cm^−1^ has shown the existence of phenolic alcoholic group in the sample. Other peak at frequency 2922.61 cm^−1^ determined the C–H bond and carboxylic acid OH. Another peak at frequency 1616.50 and 1512.39 cm^−1^ show the presence of C=C alkene and C=C aromatic compounds. The % transmittance peak at frequency of 1255.27, and 1032.85 cm^−1^ show the presence of alkyl halides. These peaks indicated the presence of phenolic and flavonoidal components. The results were related with the already performed green synthesis of FeNPs with the eucalyptus leaf extract [12,13]. The IR spectrum of ZnNPs with % transmittance peak of frequency 3345.76 cm^−1^ has shown the presence of alcoholic group of phenols. Other peak at frequency of 2916.26 cm^−1^ show the presence of C–H bond and carboxylic acid's OH. The peak at 2853.07 indicated the presence of aldehydic stretching. Another peak at frequency 1161.45 cm^−1^ has shown the presence of C–OH stretching or C–*O*–C stretching respectively. The % transmittance peak at frequency 1259.83, and 1027.08 cm^−1^ indicated the alkyl halides in the sample. These results were related with the green synthesis of ZnNPs with *Beta vulgaris*, *Cinnamomum tamala*, *Brassica oleracea* var. Italica and *Cinnamomum verum* [89]. Finally it is concluded that these present phytoconstituent were responsible for the synthesis of nanoparticles, especially polyphenolic compounds which are directly involve in the reduction of Fe and Zn ions into their zero valent state [[96], [97], [98], [99], [100]].

### 3.6 Total phenolic and flavonoids components in the crude extract

The pharmacological potential of different plant species are due to their phytochemicals which are extensively found within the plants, such as different phenolic components, flavonoids compounds or other useful secondary metabolites. These compounds play significant role in plants body as defensive agents against their environmental attacks. Total phenolic components and total flavonoids components were calculated as Gallic acid equivalent (mg GAE/g), and catechin (mg CE/g) in the extract of *P. cashmeriana* (Table-4).

**Table 4:** Total phenolic and flavonoid components present in the crude extract of *P. cashmeriana*.

Sr. no.	Evaluation of components	Quantity
1	Total phenolic components	297.51 mg GAE/g
2	Total flavonoids components	467.24 mg CE/g

### 3.7 Cytotoxicity assay

The % cytotoxicity was calculated of all the samples by following hemolytic assay and value compared with Triton X-100 positive control and PBS buffer as a negative control.

**Fig. 9:**
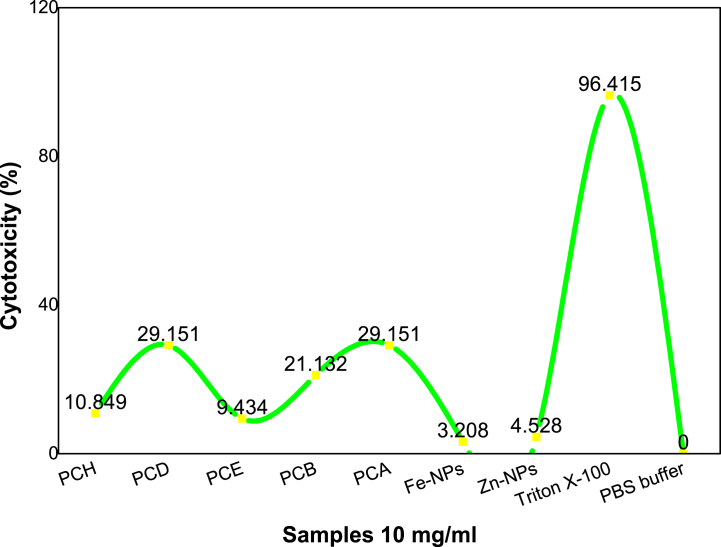
Cytotoxic potentials of different extracts of *P. cashmeriana*, Fe and Zn NPs.

Table 5 and Fig. 9 both show the toxic effects of the PCH, PCD, PCE, PCB, PCA, Fe-NPs, and Zn-NPs towards human red blood cells. The Zn-NPs and Fe-NPs show less toxicity with 4.528 % and 3.208 % values respectively. The PCE and PCH extracts have moderate toxicity with 9.343 % and 10.849 % values respectively and PCD, PCB, PCA have 29.151 %, 21.132 %, and 29.151 % toxic values towards RBCs.

**Table 5:** Cytotoxicity % of the different samples in the hemolytic assay.

Sr. no.	Type of extract	Cytotoxicity (%)	Status
1	PCH	10.849	Moderate toxic
2	PCD	29.151	Highly toxic
3	PCE	9.434	Moderate toxic
4	PCB	21.132	Highly toxic
5	PCA	29.151	Highly toxic
6	Fe-NPs	3.208	Low toxic
7	Zn-NPs	4.528	Low toxic
8	Triton X-100	96.415	Highly toxic
9	PBS buffer	0	not toxic

### 3.8 Determination of anti-inflammation activity

The anti-denaturation potentials of different extracts of *Phlomis cashmeriana* were determined by using BSA (bovine serum albumin) protein as an assay. In this assay, diclofenac was used as positive control and DMSO as negative control.

**Fig. 10:**
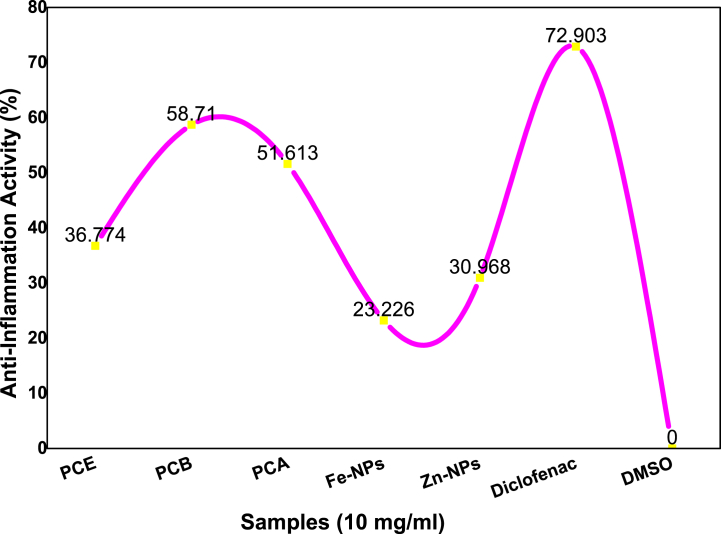
Anti-inflammatory potentials of extracts of *P. cashmeriana,* Fe and Zn NPs.

In Table 6 and Fig. 10 first two extracts PCH and PCD showed −512.258 % and −535.484 % anti-inflammatory values which clearly indicated that these two extracts are inactive against the inhibition of BSA denaturation. The PCA and PCB extracts have 58.710 % and 51.613 % values respectively, which indicated that these extracts have high anti-inflammatory potentials towards BSA. The % inhibition of PCE and Zn-NPs were 36.774 and 30.968 respectively which indicated moderate active potential and whereas the Fe-NPs have shown 23.226 % inhibition capacity against BSA protein, which indicated less active anti-inflammation potential.

**Table 6:** Percent inhibition of denaturation of tested samples.

Sr. no.	Type of extract	% Anti-inflammatory potential	Status
1	PCH	−512.258	Inactive
2	PCD	−535.484	Inactive
3	PCE	36.774	Moderate active
4	PCB	58.710	High active
5	PCA	51.613	High active
6	Fe-NPs	23.226	Less active
7	Zn-NPs	30.968	Moderate active
8	Diclofenac (positive control)	72.903	Highly active
9	DMSO (negative control)	0.00	Inactive

### 3.9 Minimum inhibitory concentration evaluation

In this method, Ciprofloxacin was used as a positive control and MIC determination was performed against two bacterial strains, i.e. *Escherichia coli* and S*taphylococcus aureus.*

#### 3.9.1 MIC against Escherichia coli

**Fig. 11:**
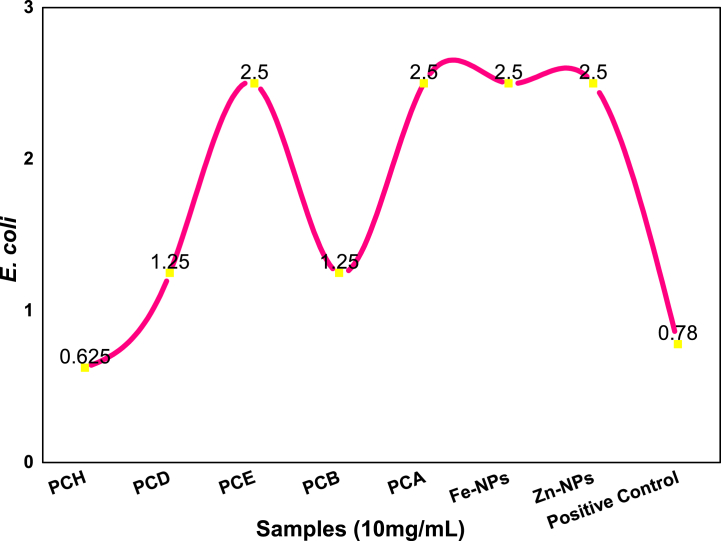
Minimum inhibitory concentration against *E. coli* against various extracts of *P. cashmeriana*.

The MIC result for *E. coli* was shown in Fig. 11. The PCH extract showed MIC value 0.625 mg/mL which was very close to the positive control. PCD and PCB extracts shown same MIC 1.25 mg/mL and PCE, PCA, Fe-, Zn-NPs have same MIC values i.e. 2.5 mg/mL. Following Table 7 shows the minimum inhibitory concentration values of all the extracts and the Fe-, Zn-nanoparticles against *E. coli* bacterial strain.

**Table 7:** Minimum inhibitory concentrations for *E. coli* bacterial strain.

Sr. no.	Type of extracts	MIC against *E. coli* (mg/mL)
1	PCH	0.625
2	PCD	1.25
3	PCE	2.5
4	PCB	1.25
5	PCA	2.5
6	Fe-NPs	2.5
7	Zn-NPs	2.5
8	Positive control	0.78

#### 3.9.2 MIC against Staphylococcus aureus

**Fig. 12:**
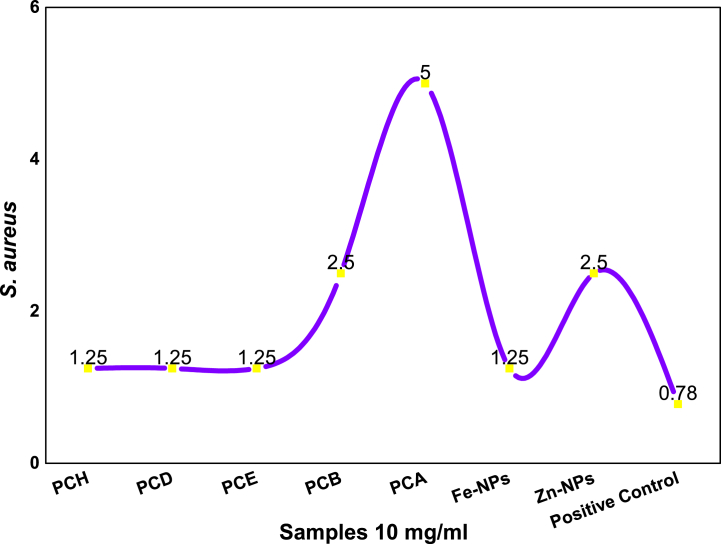
Minimum inhibitory concentration for the *S. aureus* against different extracts of *P. cashmeirana*, Fe and Zn NPs.

Fig. 12 shown the minimum inhibitory concentration of all subjected samples against *Staphylococcus aureus* bacterial strain. Three extracts PCH, PCD, PCE and Fe-NPs have the same MIC values of 1.25 mg/mL for the *S. aureus* bacterial strain. However, PCB and Zn-nanoparticles have same MIC value of 2.5 mg/mL. In the case of this bacteria, Ciprofloxacin (positive control) has same 0.78 mg/mL MIC value as shown against *S. aureus* bacterial strain in Table 8.

**Table 8:** Minimum inhibitory concentrations for *S. aureus* bacterial strain.

Sr. no.	Type of extracts	MIC against *S. aureus* (mg/mL)
1	PCH	1.25
2	PCD	1.25
3	PCE	1.25
4	PCB	2.5
5	PCA	5.0
6	Fe-NPs	1.25
7	Zn-NPs	2.5
8	Positive	0.78

The concentration of phenolic components influences the antibacterial effect most significantly; the higher the concentration, the more active the substance is. The minimum inhibitory concentration values of the different extracts of *P. cahmeriana* have shown that this plant species has great antibacterial potential.

### 3.10 Anti-thrombolytic activity

The thrombolytic activities of the extracts were evaluated by reacting them with the clots of human red blood cells. The positive control in this method was streptokinase while water was used as a negative control.

**Fig. 13:**
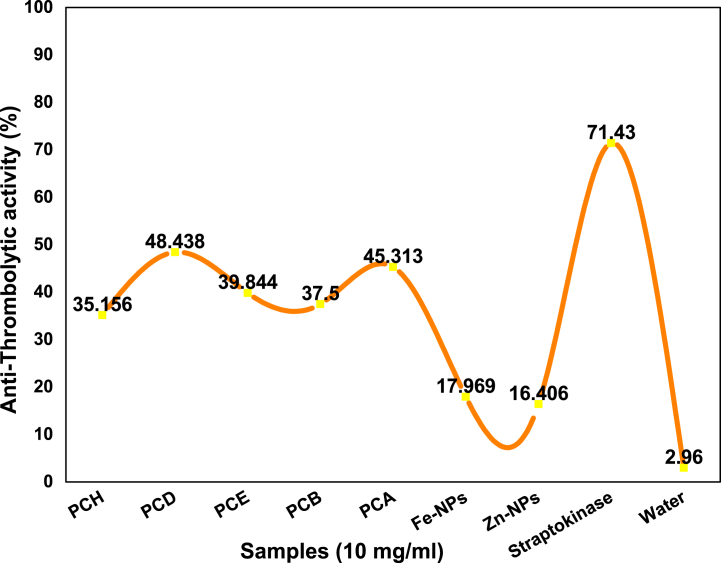
Anti-thrombolytic activity of various extracts of *P. cashmeriana*, Fe and Zn-NPs.

Table 9 and Fig. 13 showed that all the samples used in the determination of clot lysis are active in anti-thrombolytic activity. Straptokinase has clot lysis 71.43 % and whereas water has very low activity of 2.96 % towards clot lysis. PCD and PCA have high clot lysis values among the subjected samples which were 48.438 % and 45.313 % respectively. After that PCH, PCE and PCB extracts have anti-thrombolytic potential values 35.156 %, 39.844 %, and 37.500 % respectively. Fe and Zn-NPs have the lowest anti-thrombolytic potential values among all the used samples which were 17.969 % and 16.406 % respectively. Overall each extracts of the *P. cashmeriana* have active anti-thrombolytic potentials.

**Table 9:** Determination of percent clot lysis of different extracts with Fe and Zn-NPs.

Sr. no.	Samples	Eppendroff weight	Weight of tube with clot	Weigh of clot before lysis (g)	Weight of tube after lysis	Weight of clot after lysis	% Clot lysis
1	PCH	0.71	1.99	1.28	1.16	0.45	35.156
2	PCD	0.71	1.99	1.28	1.33	0.62	48.438
3	PCE	0.71	1.99	1.28	1.22	0.51	39.844
4	PCB	0.71	1.99	1.28	1.19	0.48	37.500
5	PCA	0.71	1.99	1.28	1.29	0.58	45.313
6	Fe-NPs	0.71	1.99	1.28	0.94	0.23	17.969
7	Zn-NPs	0.71	1.99	1.28	0.92	0.21	16.406
8	Straptokinase	0.71	1.99	1.28	1.62	0.91	71.43
9	Water	0.71	1.99	1.28	0.748	0.038	2.96

## 4 Conclusion

Based on the finding, this study indicated that the plant mediated synthesis of Fe and Zn NPs possess potential pharmacological applications. *Phlomis cashmeriana* contains various phytochemicals as shown in the GC-MS study, such as flavonoids, terpenoids, phenols and esters which are involved in the reduction and also assist in the formation and stabilization of these nanoparticles. The success in nanoparticle formation was observed by sudden change in color of the solutions and also with the SEM, XRD, UV and FT-IR techniques. The Folin-Ciocalteu and aluminum chloride colorimetric assays were used to determine the phenolic and flavonoids component in the extract. All extracts including Fe and Zn-NPs, overall have significant potentials in cytotoxicity, anti-bacterial, anti-inflammatory and anti-thrombolytic potentials. In hemolytic assay, Fe and Zn-NPs have less toxicity toward human red blood cells i.e. 3.208 % and 4.528 % among all other samples. Aqueous and *n*-butanol extract are shown high potential with values 51.613 % and 58.710 % against denaturing of BSA. *n*-Hexane extract has 0.625 mg/mL MIC vaues against *E. coli* which is lowest among all other subjected samples and in case of *S. aureus*; *n*-hexane, chloroform, ethyl acetate and Fe-NPs have same MIC value i.e. 1.25 mg/mL. Chloroform and aqueous extracts are shown high potential with 48.438 % and 45.313 % values in anti-thrombolytic assay. However, Fe-NPs, *n*-butanol and aqueous extracts were highly significant among all the extracts, supported by their in vitro pharmacological evaluations. As a result, it is concluded that *Phlomis cashmeriana* has a potential to be used to resist microorganism, inflammation and clotting agents with less toxicity. These properties were attributed to the availability of terpenoids, phenolic acids and flavonoids. Still, further investigation is required in the screening and identification of responsible phyto-chemicals for the complication under examination.

## Data availability statement

The authors confirm that the data supporting the findings of this study are available within the article and its supplementary materials.

## CRediT authorship contribution statement

**Amjad Hussain:** Writing – review & editing, Writing – original draft, Supervision. **Sajjad Azam:** Writing – review & editing, Writing – original draft, Visualization, Methodology, Investigation, Formal analysis, Conceptualization. **Kanwal Rehman:** Writing – review & editing, Formal analysis, Conceptualization. **Meher Ali:** Writing – review & editing, Supervision, Methodology. **Muhammad Sajid Hamid Akash:** Writing – review & editing, Investigation, Conceptualization. **Xuefeng Zhou:** Writing – review & editing, Writing – original draft, Conceptualization. **Abdur Rauf:** Methodology, Investigation, Conceptualization. **Abdulrahman Alshammari:** Writing – review & editing, Writing – original draft, Investigation, Conceptualization. **Norah A. Albekairi:** Investigation. **Abdullah Hamed AL-Ghamdi:** Methodology. **Ahmad Kaleem Quresh:** Writing – review & editing, Validation. **Shoaib Khan:** Writing – review & editing. **Muhammad Usman Khan:** Methodology.

## Declaration of competing interest

The authors declare that they have no known competing financial interests or personal relationships that could have appeared to influence the work reported in this paper.
