# Supplementary material for: Green synthesis of Fe and Zn-NPs, phytochemistry and pharmacological evaluation of Phlomis cashmeriana Royle ex Benth
Source: Heliyon. 2024 Jun 22;10(13):e33327. doi: 10.1016/j.heliyon.2024.e33327 (PMC467069; doi:10.1016/j.heliyon.2024.e33327)
Supplement: Multimedia component 1 [file mmc1.docx]

**Table 1**

The characteristics of FeNPs synthesized from the different parts of Plants.

| Plant source | Plant parts | Morphology | Size (nm) | Application | Ref. |
| --- | --- | --- | --- | --- | --- |
| *Dodonaea viscose* | Leaf | Spherical | 50-60 | Antibacterial | [[11](#_ENREF_11)] |
| Green tea and *Eucalyptus* | Leaf | Quasi-spherical | 20-80 | Nitrates removal | [[12](#_ENREF_12)] |
| *Eucalyptus* | Leaf | Amorphous | 20-80 | Treatment of eutrophic wastewater | [[13](#_ENREF_13)] |
| Green-Tea | Leaf | - | - | Soil mineralogy | [[14](#_ENREF_14)] |
| *S. jambos* (L.) Oolong tea,  *A. moluccana* (L.) | Leaf | - | - | Removal of chromium | [[15](#_ENREF_15)] |
| Green tea | Leaf | - | - | Transport properties of  nano zero-valent iron  (nZVI) through soil | [[16](#_ENREF_16)] |
| Green tea | Leaf | Spherical | 5-10 | Removal of hexavalent  chromium | [[17](#_ENREF_17)] |
| *Eucalyptus globules* | Leaf | Spherical | 50-80 | Adsorption of  hexavalent chromium | [[18](#_ENREF_18)] |
| Green tea | Leaf | Spherical | 70-80 | Degradation of dye  (malachite green) | [[19](#_ENREF_19)] |
| Green tea | Leaf | - | 20-120 | Degradation of  monochlorobenzene | [[20](#_ENREF_20)] |
| *Salvia officinalis* | Leaf | Spherical | 5-25 | - | [[21](#_ENREF_21)] |
| *Oolong tea* | Leaf | Spherical | 40-50 | Degradation of  malachite green | [[22](#_ENREF_22)] |
| *Aloe vera* | Leaf | Cubic crystalline | 6-30 | - | [[23](#_ENREF_23)] |
| Green tea | Leaf | Crystalline | 40-80 | Photo catalytic activity | [[24](#_ENREF_24)] |
| Orange extract | Peel | Cubic cystalline | 30-50 | - | [[25](#_ENREF_25)] |
| Sorghum | Bran | Spherical | 40-50 | Degradation of  bromothymol blue | [[26](#_ENREF_26)] |
| Alfalfa | - | - | 1-10 | - | [[27](#_ENREF_27)] |
| Alfalfa | - | - | ˂5 | - | [[28](#_ENREF_28)] |
| *Syzygium cumini* | Seed | Crystalline spherical | 9-20 | - | [[29](#_ENREF_29)] |
| *Passiflora tripartitavar*. | Fruit | Spherical | 18-24 | - | [[30](#_ENREF_30)] |
| *Terminalia chebula* | Fruit | Amorphous chain-like | ˂80 | - | [[31](#_ENREF_31)] |
| GarlicVine  (*Mansoa alliacea*) | Leaf | Crystalline | 13-15 | - | [[32](#_ENREF_32)] |
| *Hordeum vulgare* and  *Rumex acetosa* | Leaf | Amorphous | 10-40 | - | [[33](#_ENREF_33)] |
| *Punica granatum* | Leaf | - | 100-200 | Hexavalent chromium  removal | [[34](#_ENREF_34)] |
| *Tridax procumbens* | Leaf | Irregular sphere shape | 80-100 | Antibacterial | [[35](#_ENREF_35)] |
| *Azadirachta Indica* | Leaf | Spherical | 50-100 | - | [[36](#_ENREF_36)] |
| Carob | Leaf | Mono-dispersed crystalline | 5-8 | - | [[37](#_ENREF_37)] |
| Grape | Leaf | Amorphous quasi-Spherical | 15-100 | Azo dyes such as acid  Orange | [[38](#_ENREF_38)] |
| *Eucalyptus tereticornis*,  *Melaleuca nesophila*, and  *Rosemarinus officinalis* | Leaf | Spherical | 50-80 | Catalyst for  decolourisation of azo  dyes | [[39](#_ENREF_39), [40](#_ENREF_40)] |
| *Eucalyptus tereticornis* | Leaf | Cubic | 40-60 | Adsorption of azo dyes | [[41](#_ENREF_41)] |
| *Azadirachta indica* | Leaf | - | 100 | - | [[42](#_ENREF_42)] |
| Tea | Powder of tea | Spherical | 40-50 | - | [[43](#_ENREF_43)] |
| Green tea | Leaf | Crytalliine spherical | 70 | - | [[44](#_ENREF_44)] |
| Green tea | Leaf | Amorphous | 40-60 | Degradation of aqueous  cationic and anionic dyes | [[45](#_ENREF_45)] |
| *Camellia sinensis* | Leaf | Spherical | 5-15 | Bromothymol blue  degradation (organic  contamination) | [[46](#_ENREF_46)] |

**Table 2**

The characteristics of ZnNPs synthesized from the different parts of Plants.

| Plant source | Plant parts | Morphology | Size (nm) | Ref. |
| --- | --- | --- | --- | --- |
| *Laurus nobilis* L | Leaf | Hexagonal Wurtzite | 25-26 | [[47](#_ENREF_47)] |
| *Catharanthus roseus* | Leaf | Hexagonal Wurtzite | 50-90 | [[48](#_ENREF_48)] |
| *Cassia alata* | Leaf | Spherical | 60-80 | [[49](#_ENREF_49)] |
| *Psidium guajava* | Leaf | Spherical | 13-28 | [[50](#_ENREF_50)] |
| *Olea europaea* | Leaf | Spherical | 11-28 | [[50](#_ENREF_50)] |
| *Ficus carica* | Leaf | Spherical | 11-24 | [[50](#_ENREF_50)] |
| *Citrus limon* Osbec | Leaf | Spherical | 11-24 | [[50](#_ENREF_50)] |
| *Pandanus odorifer* | Leaf | Spherical | 90 | [[51](#_ENREF_51)] |
| *Matricaria chamomilla* L | Flowers | Crystalline | 49-191 | [[52](#_ENREF_52)] |
| *Olea europaea* | Leaf | Crystalline | 40-124 | [[52](#_ENREF_52)] |
| *Lycopersicon esculentum* M. | Fruits | Crystalline | 65-133 | [[52](#_ENREF_52)] |
| *Coccinia abyssinica* | Tuber | Hexagonal | 10.4 | [[53](#_ENREF_53)] |
| *Couroupita guianensis* | Leaf | Nanoflakes | - | [[54](#_ENREF_54)] |
| *Euphorbia jatropa* | Latex | Hexagonal | 6-21 | [[55](#_ENREF_55)] |
| *Nyctanthes arbor-tristis* | flowers | Spherical | 12-32 | [[56](#_ENREF_56)] |

**Table 3**

Chemical composition of *P. cashmeriana* methanolic extract.

| Sr. no. | R/T | Compound name | `Molecular formula | Mol. wt. | Peak area % |
| --- | --- | --- | --- | --- | --- |
| 1 | 19.984 | Silane, trimethyl [[5-methyl-2-(1-methylethyl) cyclohexyl]oxy]- | C_13_H_28_OSi | 228.45 | 0.145 |
| 2 | 20.423 | 3-Methoxy-D-homoestra-1, 3, 5(10-trien-17a-one (8-9 & 14 = α) | C_20_H_25_O_2_ | 297.43 | 2.675 |
| 3 | 24.527 | 2-Cyclopenten-1-one, 2-pentyl- | C_10_H_16_O | 152.23 | 0.754 |
| 4 | 25.910 | 1,4-Anthracenedione, 5,6,7,8-tetra hydro-2-methoxy-5,5-dimethyl- | C_17_H_18_O_3_ | 270.32 | 23.143 |
| 5 | 28.747 | 1,5,9-Cyclododecatriene, (*Z,Z,Z*)- | C_12_H_18_ | 162.27 | 48.511 |
| 6 | 29.558 | 6-Octadecenoic acid, methyl ester, (*Z*)- | C_19_H_36_O_2_ | 296.49 | 24.772 |

**Table 4**

Total phenolic and flavonoid components present in the crude extract of *P. cashmeriana*.

| Sr. no. | Evaluation of components | Quantity |
| --- | --- | --- |
| 1 | Total phenolic components | 297.51 mg GAE/g |
| 2 | Total flavonoids components | 467.24 mg CE/g |

**Table 5**

Cytotoxicity % of the different samples in the hemolytic assay.

| Sr. no. | Type of extract | Cytotoxicity (%) | Status |
| --- | --- | --- | --- |
| 1 | PCH | 10.849 | Moderate toxic |
| 2 | PCD | 29.151 | Highly toxic |
| 3 | PCE | 9.434 | Moderate toxic |
| 4 | PCB | 21.132 | Highly toxic |
| 5 | PCA | 29.151 | Highly toxic |
| 6 | Fe-NPs | 3.208 | Low toxic |
| 7 | Zn-NPs | 4.528 | Low toxic |
| 8 | Triton X-100 | 96.415 | Highly toxic |
| 9 | PBS buffer | 0 | not toxic |

**Table 6**

Percent inhibition of denaturation of tested samples.

| Sr. no. | Type of extract | % Anti-inflammatory potential | Status |
| --- | --- | --- | --- |
| 1 | PCH | -512.258 | Inactive |
| 2 | PCD | -535.484 | Inactive |
| 3 | PCE | 36.774 | Moderate active |
| 4 | PCB | 58.710 | High active |
| 5 | PCA | 51.613 | High active |
| 6 | Fe-NPs | 23.226 | Less active |
| 7 | Zn-NPs | 30.968 | Moderate active |
| 8 | Diclofenac (positive control) | 72.903 | Highly active |
| 9 | DMSO ( negative control) | 0.00 | Inactive |

**Table 7**

Minimum inhibitory concentrations for *E. coli* bacterial strain.

| Sr. no. | | Type of extracts | MIC against *E. coli* (mg/mL) |
| --- | --- | --- | --- |
| 1 | PCH | 0.625 |  |
| 2 | PCD | 1.25 |  |
| 3 | PCE | 2.5 |  |
| 4 | PCB | 1.25 |  |
| 5 | PCA | 2.5 |  |
| 6 | Fe-NPs | 2.5 |  |
| 7 | Zn-NPs | 2.5 |  |
| 8 | Positive control | 0.78 |  |

**Table 8**

Minimum inhibitory concentrations for *S. aureus* bacterial strain.

| Sr. no. | Type of extracts | MIC against *S. aureus* (mg/mL) |
| --- | --- | --- |
| 1 | PCH | 1.25 |
| 2 | PCD | 1.25 |
| 3 | PCE | 1.25 |
| 4 | PCB | 2.5 |
| 5 | PCA | 5.0 |
| 6 | Fe-NPs | 1.25 |
| 7 | Zn-NPs | 2.5 |
| 8 | Positive | 0.78 |

**Table 9**

Determination of percent clot lysis of different extracts with Fe and Zn-NPs.

| Sr. no. | | Samples | Eppendroff weight | Weight of tube with clot | Weigh of clot before lysis (g) | Weight of tube after lysis | Weight of clot after lysis | % Clot lysis |
| --- | --- | --- | --- | --- | --- | --- | --- | --- |
| 1 | PCH | | 0.71 | 1.99 | 1.28 | 1.16 | 0.45 | 35.156 |
| 2 | PCD | | 0.71 | 1.99 | 1.28 | 1.33 | 0.62 | 48.438 |
| 3 | PCE | | 0.71 | 1.99 | 1.28 | 1.22 | 0.51 | 39.844 |
| 4 | PCB | | 0.71 | 1.99 | 1.28 | 1.19 | 0.48 | 37.500 |
| 5 | PCA | | 0.71 | 1.99 | 1.28 | 1.29 | 0.58 | 45.313 |
| 6 | Fe-NPs | | 0.71 | 1.99 | 1.28 | 0.94 | 0.23 | 17.969 |
| 7 | Zn-NPs | | 0.71 | 1.99 | 1.28 | 0.92 | 0.21 | 16.406 |
| 8 | Straptokinase | | 0.71 | 1.99 | 1.28 | 1.62 | 0.91 | 71.43 |
| 9 | Water | | 0.71 | 1.99 | 1.28 | 0.748 | 0.038 | 2.96 |

**
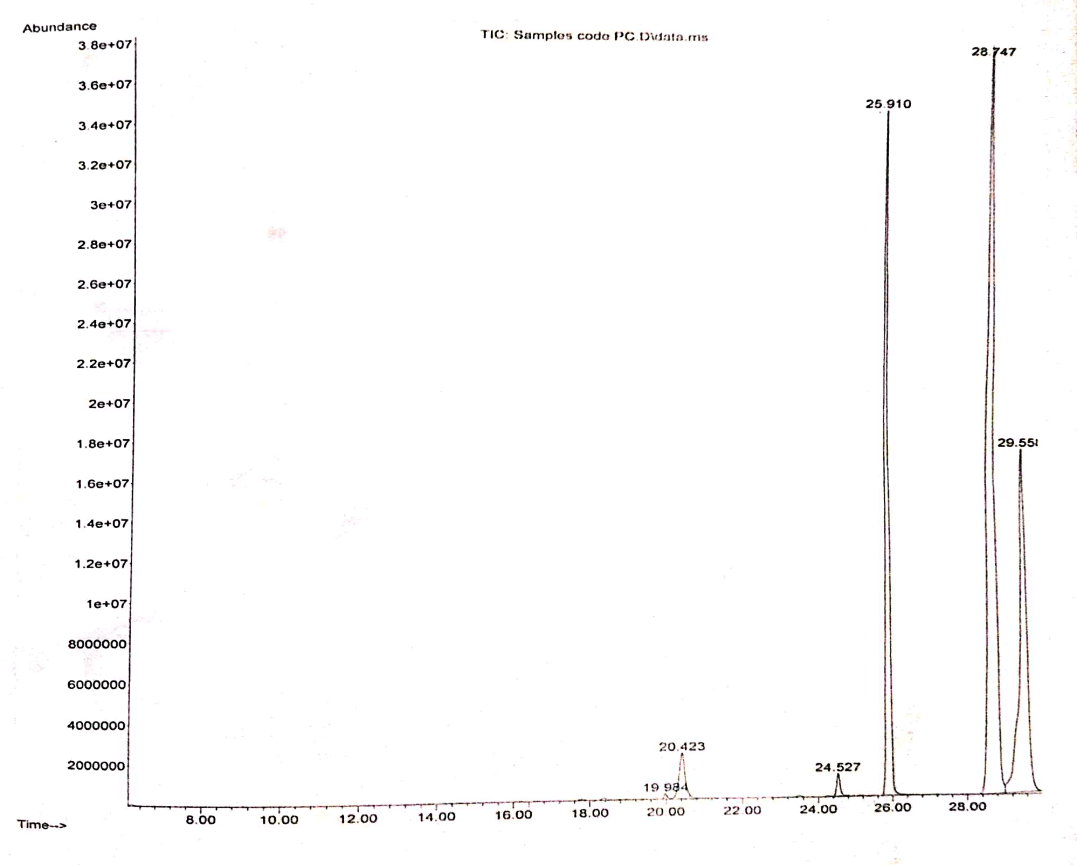
**

**Fig. 1.** GC-MS analysis report for the extract of *P. cashmeriana*.

**
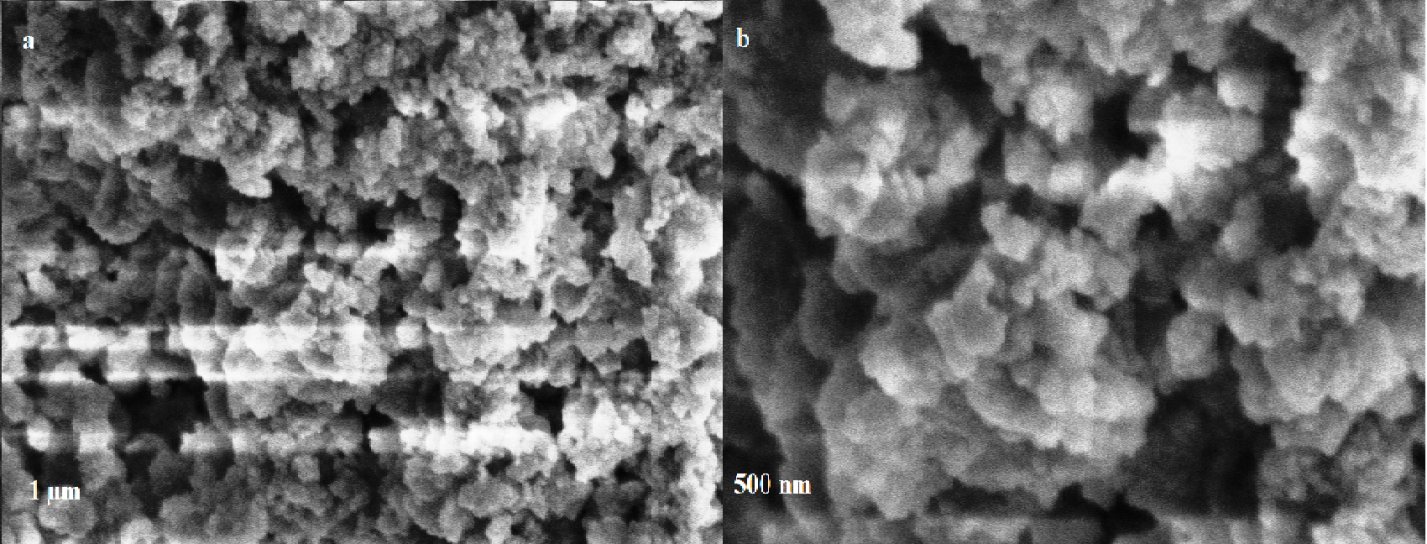
**

**Fig. 2.** SEM images of Fe-NPs; a) low magnification, b) high magnification.

 **Fig. 3.** SEM images of Zn-NPs; a) low magnification, b) high magnification.

**Fig. 4.** UV data for the crude extract (PC) and synthesized Fe-NPs. NP 1a: PC solution and salt solution in 1:1 v/v, NP 1b: PC solution and salt solution in 1:2 v/v

**Fig. 5.** UV data for the crude extract (PC) and synthesized Zn-NPs, NP 2a: PC solution and salt solution in 1:1 v/v, NP 2b: PC solution and salt solution in 1:2 v/v

**Fig. 6.** XRD pattern for the green synthesize Fe-NPs.

**Fig. 7.** XRD pattern for the green synthesize Zn-NPs.

**Fig.8.** FT-IR spectrum of crude extract of *P. cashmeriana* (PC), FeNPs, and ZnNPs.

**Fig. 9.** Cytotoxic potentials of different extracts of *P. cashmeriana*, Fe and Zn NPs.

**Fig. 10.** Anti-inflammatory potentials of extracts of *P. cashmeriana,* Fe and Zn NPs.

.

**Fig. 11.** Minimum inhibitory concentration against *E. coli* against various extracts of *P. cashmeriana*.

**Fig. 12.** Minimum inhibitory concentration for the *S. aureus* against different extracts of *P. cashmeirana*, Fe and Zn NPs.

**Fig. 13.** Anti-thrombolytic activity of various extracts of *P. cashmeriana*, Fe and Zn-NPs.
